# Leptin Induces Oncostatin M Production in Osteoblasts by Downregulating miR-93 through the Akt Signaling Pathway

**DOI:** 10.3390/ijms150915778

**Published:** 2014-09-05

**Authors:** Wei-Hung Yang, Chun-Hao Tsai, Yi-Chin Fong, Yuan-Li Huang, Shoou-Jyi Wang, Yung-Sen Chang, Chih-Hsin Tang

**Affiliations:** 1 Department of Orthopedic Surgery, Taichung Hospital, Department of Health Executive Yuan, Taichung 403, Taiwan; E-Mails: u766018@ms42.hinet.net (W.-H.Y.); wendy793219@yahoo.com.tw (Y.-S.C.); 2 Department of Nursing, National Taichung University of Science and Technology, Taichung 403, Taiwan; 3 School of Chinese Medicine, College of Chinese Medicine,China Medical University, Taichung 404, Taiwan; E-Mail: yichin.fong@gmail.com; 4 Graduate Institute of Biotechnology, National Chung Hsing University, Taichung 402, Taiwan; 5 Department of Medicine and Graduate Institute of Clinical Medical Science, China Medical University, Taichung 404, Taiwan; E-Mail: ritsai8615@gmail.com; 6 Department of Orthopedic Surgery, China Medical University Hospital, Taichung 404, Taiwan; 7 Department of Biotechnology, College of Health Science, Asia University, Taichung 500, Taiwan; E-Mail: yuanli@asia.edu.tw; 8 Department of Orthopedic Surgery, Chang-Hua Hospital, Department of Health Executive Yuan, Changhua Country 500, Taiwan; E-Mail: sdsaw@msn.com; 9 Graduate Institute of Basic Medical Science, China Medical University, Taichung 404, Taiwan; 10 Department of Pharmacology, School of Medicine, China Medical University, Taichung 404, Taiwan

**Keywords:** leptin, oncostatin M, OBRl, arthritis, miR-93

## Abstract

Inflammatory response and articular destruction are common symptoms of osteoarthritis (OA) and rheumatoid arthritis (RA). Leptin, an adipocyte-secreted hormone that centrally regulates weight control, may exert proinflammatory effects in the joint, depending on the immune response. Yet, the mechanism of leptin interacting with the arthritic inflammatory response is unclear. This study finds that leptin increased expression of oncostatin M (OSM) in human osteoblasts in a concentration- and time-dependent manner. In addition, OBRl, but not OBRs receptor antisense oligonucleotide, abolished the leptin-mediated increase of OSM expression. On the other hand, leptin inhibited miR-93 expression; an miR-93 mimic reversed leptin-increased OSM expression. Stimulation of osteoblasts with leptin promoted Akt phosphorylation, while pretreatment of cells with Akt inhibitor or siRNA reversed leptin-inhibited miR-93 expression. Our results showed that leptin heightened OSM expression by downregulating miR-93 through the Akt signaling pathway in osteoblasts, suggesting leptin as a novel target in arthritis treatment.

## 1. Introduction

Arthritis as a systemic inflammatory process comprises osteoarthritis (OA) and rheumatoid arthritis (RA), which leads to joint destruction and extra articular symptoms, with a significant effect on morbidity and mortality [1,2,3]. As cartilage impaired or monocytes infiltrated the synovium, proinflammatory cytokines were secreted during the development of arthritis, which caused synovial hyperplasia, secretion of degradative enzymes and long-term bone erosion and damage [4,5]. A previous study showed that chemokines were released directly or indirectly from subchondral bone, which caused bone remodeling and cartilage destruction in arthritis [6]. As cartilage was depreciated in arthritis pathogenesis, some studies indicated that subchondral bone also played a key role in OA and RA process [7,8]. Hence, subchondral bone potentially acts in concert as a mechanical environment for development of arthritis.

Oncostatin M (OSM), 28 kDa, a cytokine of the interleukin-6 (IL-6) family, is multifunctional (skeletal tissue alteration, bone metabolism, inflammatory disease) and originates from monocytes, macrophages or T-cells within chronic inflammatory processes [5,9,10]. Studies indicated that OSM is omnipresent in synovial fluid and serum in OA and RA cases [11,12,13], resulting in the secretion of proinflammatory cytokines TNF-α, IL-1β and IL-6 from osteoblasts and synovial cells, which degrade cartilage in arthritic joints [14,15,16], hinting at OSM’s role in pathogenesis. Leptin, a small (16 kDa) nonglycosylated peptide hormone encoded by the obese (ob) gene [17], is produced predominantly in white adipose tissue [18]. Leptin is an anorexic peptide that is primarily known for its role as a hypothalamic modulator of food intake, body weight and fat stores [19]. The biological activity of leptin is mediated by specific receptors (OBR), which are located in several tissues throughout the body [20]. At least six isoforms of OBR are generated by alternative messenger RNA splicing, but in humans, two major forms of leptin receptor are expressed. The long form of the receptor (OBRl), which is believed to be the signaling-competent receptor isoform, is essential in mediating most of the biological effects of leptin [21]. The signaling pathways activated by OBRl include the classic cytokine JAK2/STAT3 pathway, as well as the insulin receptor substrate (IRS)-1/PI3K/Akt pathway [22]. On the other hand, the microRNAs (miRNAs) are small (about 22-nucleotides long), non-coding RNAs that can modulate targeted gene expression through either translational repression or mRNA cleavage. miRNAs have been indicated to regulate inflammatory cytokine production [23]. In addition, miR-93 has been reported to be a negative regulator of the immune response [24]. Although some molecular targets are documented, the role of miR-93 in OSM expression is largely unknown.

Past research showed arthritis correlating with osteoclast differentiation, and a recent study indicates that osteoblasts also participate in the inflammation process [25,26], OSM being strongly expressed in osteoblasts isolated from femora in arthritics [6,26]. OSM can regulate arthritis associated with osteoblasts [16,27]. The effect of leptin-induced OSM expression in osteoblasts is yet unclarified. This study investigated the signal pathway-involved, leptin-induced OSM production in human osteoblasts. The results show that leptin increases OSM expression by downregulating miR-93 through the Akt signaling pathway.

## 2. Results

### 2.1. Leptin Induces OSM Expression in Human Osteoblasts through the OBRl Receptor

**Figure 1:**
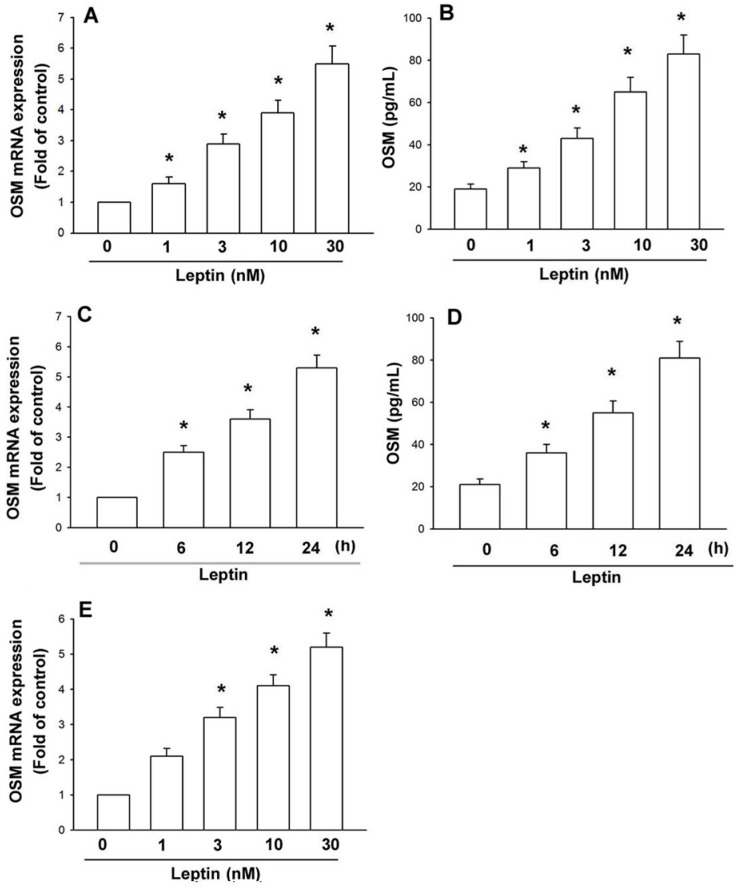
Leptin induces OSM expression in human osteoblasts. (**A**,**B**) Osteoblasts were incubated with various concentrationsof leptin for 24 h.Media and total RNA were collected, and the expression of OSM was examined by qPCR and ELISA assay (*n* = 5); (**C**,**D**) Osteoblasts were incubated with leptin (30 nM) for 6, 12 or 24 h. Media and total RNA were collected, and the expression of OSM was examined by the qPCR and ELISA assay (*n* = 4); (**E**) Osteoblasts from OA patients were incubated with various concentrationsof leptin for 24 h.Total RNA was collected, and the expression of OSM was examined by qPCR (*n* = 3). The results are expressed as the mean ± SEM; ^*^ *p* < 0.05 as compared with the basal level.

**Figure 2:**
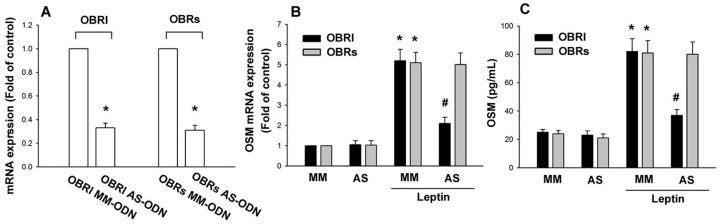
Leptin induces OSM expression through the OBRl receptor. (**A**) Osteoblasts were transfected with OBRl and OBRs antisense oligonucleotide (AS-ODN) or OBRl and OBRs missense (MM)-ODN, and the mRNA level of OBRl and OBRs was analyzed by qPCR (*n* = 5); (**B**,**C**) Osteoblasts were transfected with OBRl and OBRs AS-ODN or OBRl and OBRs MM-ODN for 24 h and then stimulated with leptin (30 nM) for 24 h; OSM expression was examined by the qPCR and ELISA assay (*n* = 5). Results are expressed as the mean ± SEM; ^*^ *p* < 0.05 as compared with the basal level; ^#^ *p* < 0.05 as compared with the leptin-treated group.

Leptin is significantly higher in the synovial fluid of patients with OA and RA [28,29]. Osteoblasts play a vital role in arthritis by producing inflammatory cytokines. We used human osteoblasts to investigate the signaling pathways of leptin in the production of OSM. Treatment of osteoblasts with leptin (1–30 nM) for 24 h induced *OSM* mRNA expression in a concentration-dependent manner (Figure 1A). Leptin stimulation meant a concentration-dependent rise in OSM protein expression, as highlighted by ELISA (Figure 1B); this induction occurred in a time-dependent manner (Figure 1C,D). We also used osteoblasts from OA patients to confirm the role of leptin. The result also indicated that leptin increased *OSM* expression in OA osteoblasts (Figure 1E). These data suggest that leptin increases OSM expression in osteoblasts. Previous studies have shown that leptin exerts its cell functions through interaction with specific leptin receptors OBRl and OBRs [30]. We therefore hypothesized that leptin receptors may be involved in leptin-mediated OSM expression in osteoblasts. Transfection with OBRl or OBRs antisense oligonucleotide (AS-ODN) specifically inhibited OBRl or OBRs expression, respectively (Figure 2A). In addition, OBRl AS-ODN, but not with OBRl missense (MM)-ODN, OBRs AS-ODN or OBRs MM-ODN, abolished the leptin-induced OSM production (Figure 2B,C). Therefore, the OBRl receptor plays a key role in leptin-induced OSM expression in osteoblasts.

### 2.2. Leptin Increases OSM Production in Osteoblasts by Inhibition of miR-93 Expression

**Figure 3:**
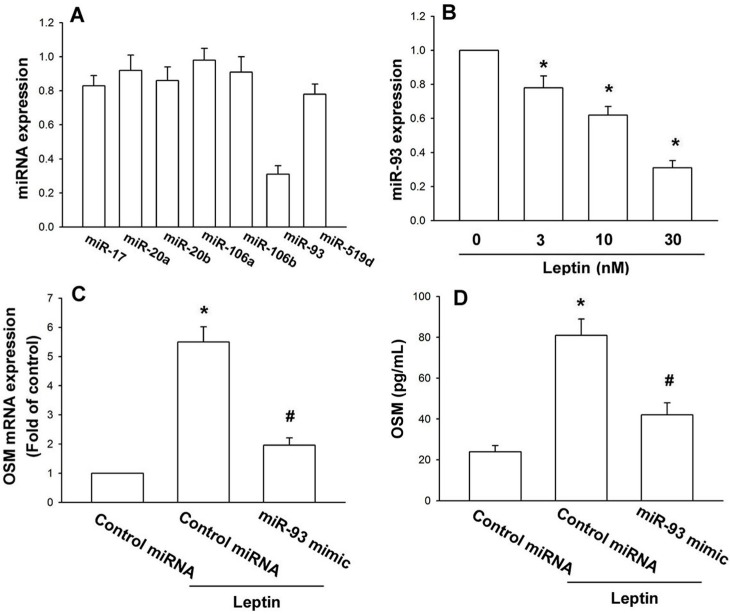
Leptin increases OSM expression through inhibition of miR-93 expression.(**A**) Osteoblasts were incubated with leptin (30 nM) for 24 h; the miRNAs’ expression was assessed by qPCR; (**B**) Osteoblasts were incubated with leptin for 24 h; miR-93’s expression was assessed by qPCR; (**C**,**D**) Osteoblasts were transfected with the miR-93 mimic for 24 h, followed by stimulation with leptin (30 nM) for 24 h; OSM expression was examined by the qPCR and ELISA assay (*n* = 5). The results are expressed as the mean ± SEM; ^*^ *p* < 0.05 as compared with the basal level; ^#^ *p* < 0.05 as compared with the leptin-treated group.

miRNAs have been reported as important regulators of inflammatory cytokines production [31,32]. We hypothesized that miRNA may regulate leptin-mediated OSM expression, using online computational algorithms (TargetScan) and filtering out seven candidate miRNAs that target *OSM*, to find that miR-93 was mostly downregulated by leptin treatment (Figure 3A); leptin concentration-dependently decreased miR-93 expression (Figure 3B). To affirm miR-93 involvement in leptin-increased OSM production, an miR-93 mimic of cells reversed leptin-increased *OSM* mRNA and protein expression (Figure 3C,D). Data suggest that leptin increases OSM production by inhibiting miR-93 expression.

### 2.3. Leptin Increases OSM Expression through the Akt Signaling Pathway

**Figure 4:**
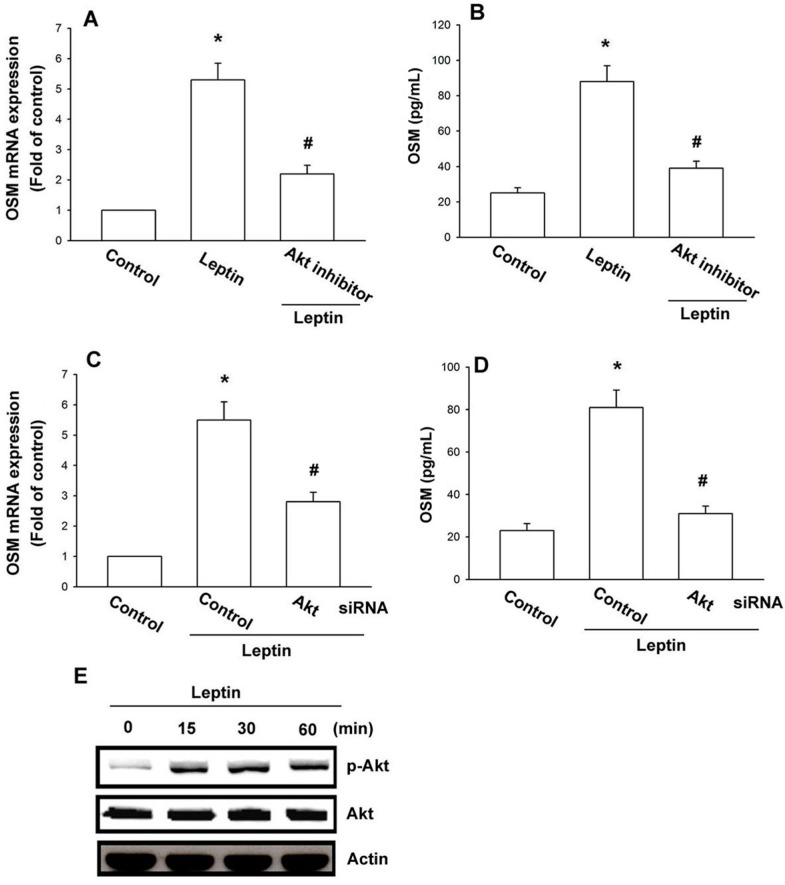
Leptin increases *OSM* expression through the Akt pathway in osteoblasts. (**A**–**D**) Osteoblasts were pretreated with Akt inhibitor (10 µM) for 30 min or transfected with Akt siRNA for 24 h followed by stimulation with leptin (30 nM) for 24 h; *OSM* expression was examined by the qPCR and ELISA assay; (**E**) Osteoblasts were incubated with leptin (30 nM) for the indicated time intervals, Akt phosphorylation was examined by western blotting. Results are expressed as the mean ± SEM; ^*^ *p* < 0.05 as compared with the basal level; ^#^ *p* < 0.05 as compared with the leptin-treated group.

**Figure 5:**
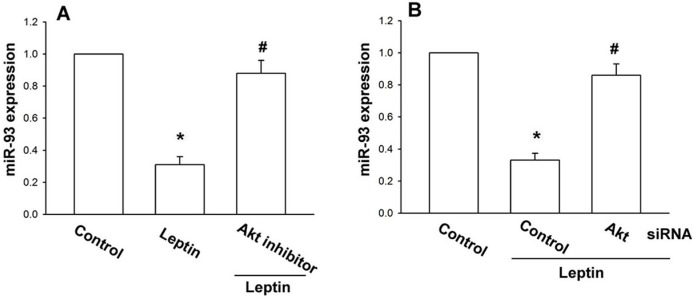
Leptin increases OSM expression by inhibition miR-93 through the Akt pathway.Osteoblasts were pretreated with Akt inhibitor (10 µM) (**A**) for 30 min or transfected with Akt siRNA (**B**) for 24 h followed by stimulation with leptin (30nM) for 24 h; miR-93 expression was measured by qPCR. Results are expressed as the mean ± SEM; ^*^ *p* < 0.05 as compared with the basal level; ^#^ *p* < 0.05 as compared with the leptin-treated group.

Previous studies have shown that leptin induced Akt activity to regulate cell functions [33,34,35]. After the stimulatory effect of leptin on *OSM* expression was revealed, its effects on the expression of the Akt pathway were assessed. Treatment with Akt inhibitor or transfection with Akt siRNA significantly counteracted leptin-increased *OSM* expression (Figure 4A–D); incubation of cells with leptin enhanced Akt phosphorylation time-dependently (Figure 4E). Letpin seems to act through a signaling pathway involving Akt to promote *OSM* expression in human osteoblasts. We tested to see if Akt is upstream in leptin-inhibited miR-93 expression. Treatment with the Akt inhibitor or transfection of Akt siRNA reversed leptin-inhibited miR-93 expression (Figure 5), *i.e.*, leptin increases *OSM* production by inhibiting miR-93 expression via the Akt signaling pathway.

## 3. Discussion

Arthritis is a heterogeneous group of conditions that are associated with the defective integrity of articular cartilage. The chronic inflammatory process is mediated through a complex cytokine network. Since OSM is constitutively expressed in the bone compartment and detected in patients with arthritis pathology [26], using OSM antibody could decrease cartilage destruction of knee joints *in vivo* [36]. This study identified OSM as a target protein for regulation of cell inflammatory response. We also showed the potentiation of OSM activated by leptin through inhibiting miR-93 expression via Akt signal pathway in osteoblasts. It has been reported that the concentration of leptin in the synovial fluid of OA patients is ~20.77 ng/mL (~1.2 nM) [28]. In the current study, we found that leptin 1 nM only slightly increased OSM expression in human osteoblasts (Figure 1). Therefore, the *in vivo* pathologic condition is more complicated than the *in vitro* culture system.

The leptin receptor belongs to the cytokine receptor superfamily [37]. Recent studies have demonstrated higher levels of leptin receptors OBRl and OBRs in the synovial fluid [28] and in the cartilage [30] of individuals with OA and RA. It has been reported that leptin receptor expression is significantly elevated in advanced arthritic cartilage compared to minimally-affected cartilage [38]. The results of this study showed that transfection with OBRl AS-ODN, but not with OBRs AS-ODN, antagonized the leptin-induced OSM production. These results suggest that OBRl is an upstream receptor in leptin-induced OSM production in human osteoblasts.

Akt is a cytoplasmic serine kinase that is important in regulating cell growth, differentiation, adhesion and inflammatory reactions [39,40]. Akt activation is also reported as regulating OSM expression [41]. In the current study, we showed leptin inducing Akt phosphorylation, while the Akt inhibitor or siRNA antagonized the leptin-mediated potentiation of OSM expression in osteoblasts, suggesting Akt activation as *sine qua non* in leptin-induced OSM production by osteoblasts. Suppressor of cytokine signaling (SCOS) proteins also play a key role in leptin signaling [42,43]. In the current study, we did not examine the role of SCOS in leptin-induced OSM expression in human osteoblasts. Whether SCOS also mediated leptin-increased OSM expression in osteoblasts needs further examination.

Newly identified, small noncoding miRNAs belong to a novel class of regulators that control gene expression by binding to complementary sequences in 3’UTRs of target mRNAs [44,45]. We hypothesized that miRNA mediated leptin-increased *OSM* production, finding that leptin decreased miR-93 expression most, but only slightly affecting the expression of miR-17, -20a, -20b, -106a, -106b and -519d, which target *OSM*. We also used a miR-93 mimic to confirm the role of miR-93, finding diminished leptin-enhanced *OSM* expression. By contrast, cell incubation with the Akt inhibitor or siRNA abolished leptin-reduced miR-93 expression. These indicate that leptin increased OSM yield by inhibiting miR-93 expression through the Akt pathway.

## 4. Experimental Section

### 4.1. Materials

We obtained control miRNA, the miR-93 mimic and Lipofectamine 2000 from Life Technologies (Carlsbad, CA, USA), as well as the Akt inhibitor (sc-394003), rabbit polyclonal antibodies for p-Akt and Akt; both Akt and the control siRNA were purchased from Santa Cruz Biotechnology (Santa Cruz, CA, USA). The recombinant human leptin and OSM ELISA kit were purchased from PeproTech (Rocky Hill, NJ, USA). All other chemicals were purchased from Sigma-Aldrich (St. Louis, MO, USA).

### 4.2. Cell Culture

Human primary osteoblasts were obtained from Lonza (Walkersville, MD, USA), and the cells were maintained at 37 °C in 5% CO2 atmosphere in RPMI-1640 medium supplemented with 20 mM HEPES, 10% heat-inactivated FBS, 2 mM glutamine, 100 U/mL penicillin and 100 μg/mL streptomycin (Invitrogen, Carlsbad, CA, USA).

### 4.3. Measurement of OSM Production

Human osteoblasts were cultured in 24-well culture plates. At confluence, cells were treated with leptin and then incubated in a humidified incubator at 37 °C for 24 h. For the examination of the downstream signaling pathways involved in leptin treatment, cells were pretreated with various inhibitors for 30 min or transfected with the miRNA mimic or siRNA for 24 h before leptin (30 nM) administration. After incubation, the medium was removed and stored at −80 °C until the assay. *OSM* in the medium was assayed using the *OSM* enzyme immunoassay kits, according to the procedure described by the manufacturer.

### 4.4. Real-Time Quantitative PCR of mRNA and miRNA

Total RNA was extracted from osteoblasts by a TRIzol kit (MDBio, Taipei, Taiwan). Reverse transcription proceeded with 1 μg of total RNA and oligo(dT) primer [46]. The real-time quantitative PCR (RT-qPCR) analysis used the Taqman^®^ one-step PCR Master Mix (Applied Biosystems, Foster City, CA, USA); 100 ng of total cDNA were added per 25 μL reaction with sequence-specific primers and Taqman^®^ probes. Sequences for target gene primers and probes were purchased commercially (GAPDH as the internal control) (Applied Biosystems, Foster City, CA, USA). The qPCR assays were carried out in triplicate by a StepOnePlus sequence detection system. Cycling conditions consisted of 10-min polymerase activation at 95 °C followed by 40 cycles at 95 °C for 15 s and 60 °C for 60 s. The threshold was set above the non-template control background and within linear phase of the target gene amplification to calculate the cycle number at which the transcript was detected (denoted as *C*_t_) [47].

For the miRNA assay, cDNA was synthesized from total RNA (100 ng) by the TaqMan MicroRNA Reverse Transcription Kit (Applied Biosystems, Foster City, CA, USA); reactions were incubated first at 16 °C for 30 min, then at 42 °C for 30 min, followed by inactivation at 85 °C for 5 min. Reactions were incubated in a 96-well plate at 50 °C for 2 min, 95 °C for 10 min, followed by 30 cycles of 95 °C for 15 s and 60 °C for 1 min by a StepOnePlus sequence detection system. Relative quantification of gene expression was performed with an endogenous control gene (U6), the threshold cycle (*C*_t_) defined as the fractional cycle number at which fluorescence passed the fixed threshold. Relative expression was calculated by the comparative *C*_t_ method.

### 4.5. Western Blot Analysis

Cellular lysates were prepared, proteins resolved by SDS-PAGE [48,49] and transferred to Immobilon polyvinylidene fluoride membranes. Blots were blocked with 4% bovine serum albumin for 1 h at room temperature, then probed with rabbit anti-human antibodies against p-Akt, Akt or Actin (1:1000) for 1 h at room temperature (Santa Cruz, CA, USA). After three washes, blots incubated with peroxidase-conjugated donkey anti-rabbit secondary antibody (1:1000) for 1 h at room temperature were visualized with enhanced chemiluminescence, using X-OMAT LS film (Eastman Kodak, Rochester, NY, USA).

### 4.6. Synthesis of OBRl and OBRs Decoy Oligonucleotide

We used a phosphorothioate double-stranded decoy oligonucleotide (ODN) carrying the OBRl antisense ODN (AS-ODN; AGACCGAGCGGGCGTTAA) and missense ODN (MM-ODN; AGCCCGCGCGAGTGTTCA) (GenBank Accession No. U43168) and the OBRs AS-ODN (TTGTCTTGCCGACCACCA) and MM-ODN (TTATCTTACCAACCGCCA) (GenBank Accession No. U50748). ODN (5 μM) was mixed with Lipofectamine 2000 (10 μg/mL) for 30 min at room temperature, and the mixture was added to cells in serum-free medium. After 24 h of transient transfection, the cells were used for the following experiments.

### 4.7. Statistical Analysis

Data were expressed as the means ± SE. Statistical analysis was performed with GraphPad Prism 4. Analysis of variance (ANOVA) and an unpaired two-tailed Student’s *t-*test were used to determine the significant differences between the means. *p* < 0.05 was considered significant.

**Figure 6:**
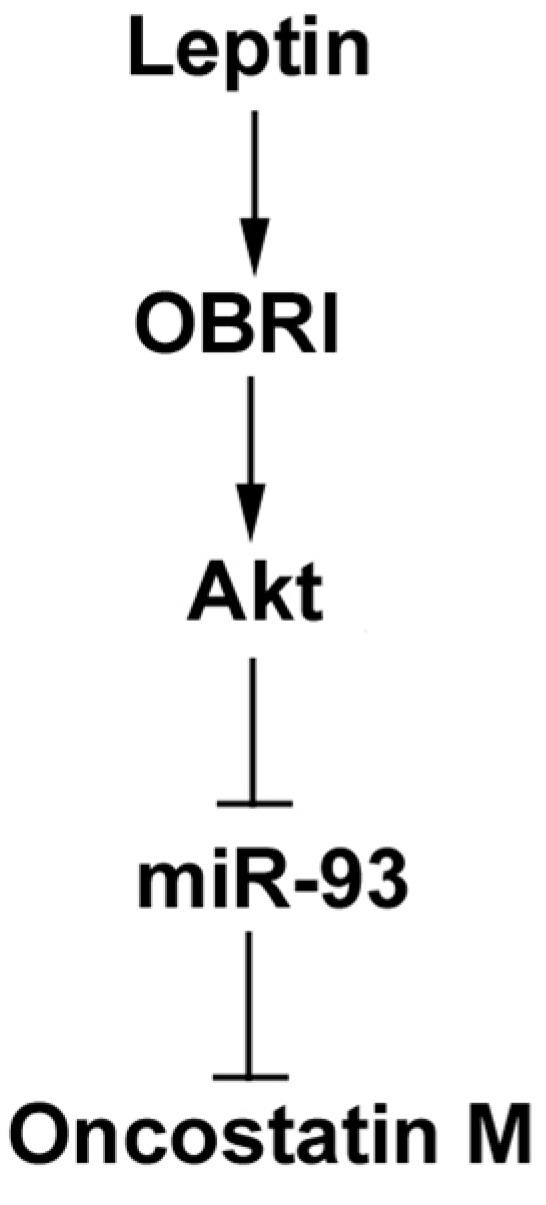
Schema of signaling pathways involved in leptin-induced OSM expression in osteoblasts.Leptin enhances OSM production in human osteoblasts by inhibition miR-93 expression through the Akt signaling pathway.

## 5. Conclusions

OA and RA are degenerative joint disease characterized by cartilage breakdown, the formation of bony outgrowths at the joint margin (osteophytes), subchondral bone sclerosis and alterations to the joint capsule [50]. Subchondral bone potentially acts in concert as a mechanical environment for development of arthritis. In this study, we investigated the signaling pathway involved in leptin-induced OSM production in human osteoblasts. The results showed that leptin increases OSM production by binding to the OBRl receptor and activating Akt signaling, which reduces miR-93 expression and leads to the transactivation of OSM production (Figure 6).
